# Mucoromycosis and Wegner’s Granulomatosis: A Case Report

**Published:** 2018-11

**Authors:** Nasrin SHAYANFAR, Abbas ZAMANIAN, Elham BEHRANGI, Nahid KIANMEHR, Faezeh FIROUZI, Zahra AZIZIAN

**Affiliations:** Dept. of Pathology, Rasoul-e-Akram Hospital, Iran University of Medical Sciences, Tehran, Iran

**Keywords:** Mucoromycosis, Wegner’s granulomatosis, Case report, Iran

## Abstract

Wegener’s granulomatosis is a rare vasculitis affecting the upper and lower respiratory tracts and kidneys. The cornerstone of treatment in these patients is immunosuppressive therapy, which may predispose the patient to super-infections such as fungal diseases per se. However, the fungal infection mimics the clinical manifestations of Wegener’s diseases would lead to neglected course of the infection and subsequent morbidity and mortality especially if unusual organs are involved. Here we report a 21-yr-old female patient referred to a hospital, Tehran, Iran in 2013 with a neglected skin mucormycosis and the course of the disease and outcome with Wegener’s granulomatosis.

## Introduction

Mucormycosis cause acute, invasive and frequently fatal infections in susceptible patients. The most common clinical forms of mucormycosis were rhinocerebral, pulmonary, and cutaneous. Rhizopus species were the most prevalent, followed by Mucor species (1). The majority of patients developing mucormycosis reported have immune deficiency. High mortality rate of advanced mucormycosis, early diagnosis and treatment may significantly improve survival rates. Wegener’s granulomatosis is a rare vasculitis of small and medium-sized vessels affecting mainly the upper and lower respiratory tracts and kidneys (2). The clinical presentations generally include cough, hematuria, hemoptysis, and other organ-related manifestations (3, 4).

The patients are usually treated with a combination of corticosteroids and cyclophosphamide or other immunosuppressors (such as methotrexate and mycophenolate mofetil) (5, 6). This matter would result in increased rate of opportunistic infections (7, 8). Mucormycosis is an example of such fungal infections. However, the skin involvement is rare (9). A diagnosis of mucormycosis was established, Based on the clinical, radiological, and histopathological study definitive diagnosis is according to the biopsy and culture of suspicious tissues (10). Correct diagnosis would result in prompt anti-fungal therapy and modification of immuno-suppressor regimens accompanying with removal surgery (8). However, the fungal infection mimics the clinical manifestations of Wegener’s diseases leading to neglected course of the infection and subsequent morbidity and mortality especially if unusual organs be involved (11).

In this paper, we report a case of neglected skin mucormycosis and the course of the disease and outcome in a patient with Wegener’s granulomatosis.

## Case Presentation

**Fig. 1:**
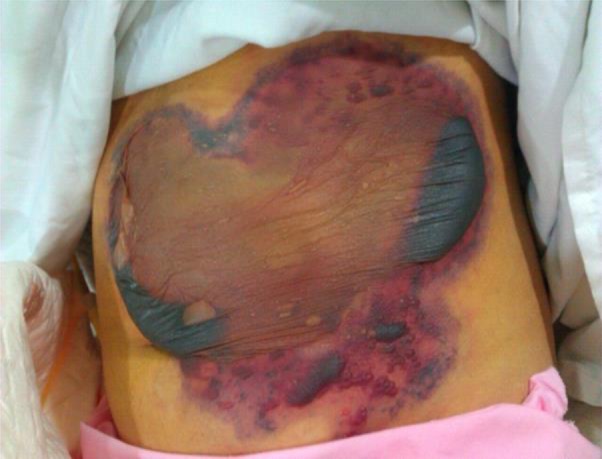
Erythematous plaque with hemorrhagic bullous lesion on abdomen

A 21-year-old female patient was referred to a general training hospital, Tehran, Iran in 2013 due to cough and skin lesions initiating from lower abdomen spreading to medial part of right shin. The cough and rhinorrhea were begun since 20 d ago and two weeks later the erythematous plaque with hemorrhagic bulla was presented in lower abdomen (Fig. 1). Three days after admission, the skin lesions were extended and the abdominal pain was initiated.

The study was approve by Ethics Committee of Iran University of Medical Sciences, Tehran, Iran. The patient had known as Wegener’s granulomatosis since four years ago. She also had anemia, arthritis, and arthralgia. She has also admitted two years ago for hematuria and hemoptysis. Patient had negative history for smoking, addiction, diabetes mellitus, and allergy. Nevertheless, anemia was present in her with a suspicious bone marrow aspiration sample.

The patient was receiving mycophenolate mofetil (500 mg three times a daily, cotrimoxazole (two tablets at bedtime) and methylprednisolone (20 mg three times a day). The findings in laboratory tests were as below; hemoglobin 8 gr/dl, BUN 34, creatinin 2.1, positive CRP, ESR 90 mm/hour, positive Anti-PR3 and negative ANA, positive blood culture for Staphylococcus aureus, hematuria, proteinuria, and glycosuria.

**Fig. 2:**
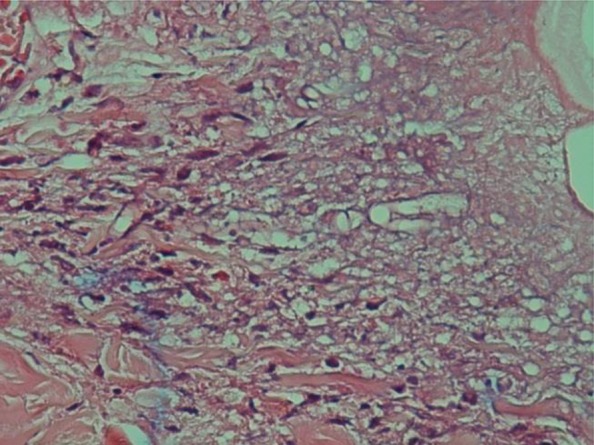
Mucor hyphae in the vessel wall and necrotic tissue (The H & E staining of the skin lesion (×100)

During recent admission, the skin lesions and productive cough were developed. However, the vital signs were normal. There was a low-grade fever. A fine crackle was heard at upper chest considered because of Wegener’s granulomatosis-related cavity in lung initially seen in at first admission. The ulcerative skin lesions and accompanied ecchymosis were seen at lower abdomen and right shin. These were expanded after corticosteroid and cyclophosphamide therapy. Histological examination of deep incisional biopsies of skin lesion revealed infiltrating lymphocytes, neutrophils, multinucleated giant cells. Hematoxylineosin (H&E) and periodic acid Schiff (PAS) staining showed numerous broad, aseptate and irregularly branched fungal hyphae indicative of mucormycosis deposited within the hypoderm and vessel wall (Fig. 2). Ten days after admission acute respiratory distress was developed leading to admission in intensive care unit. After 24 h, the patient was expired due to cardiopulmonary arrest and 45 min cardiopulmonary resuscitation was ineffective. The final CT-scan revealed extensive alveolar hemorrhage.

## Discussion

The presented case in this paper was a patient with Wegener’s granulomatosis manifesting with progressive fungal skin lesions due to mucormycosis. Despite four-year slow-progressive Wegener’s granulomatosis, rapid presentation of skin lesions and sudden acute respiratory distress leading to expiration was an unusual manifestation. On the other hand, skin mucormycosis as the cause of rapid progression is a rare phenomenon in patients with Wegener’s granulomatosis. Mucormycosis can mimic the relapse phase of Wegener’s granulomatosis (11). A known case of Wegener’s granulomatosis, with sinus mucormycosis, successfully was treated with amphotericin B surgical debridement, and intravenous immunoglobulin (12). However, in their study, the patient was not neglected. In addition, four infectious cases were diagnosed in patients with Wegener’s granulomatosis and they also were not neglected. However, so far has no case of Wegener’s granulomatosis with skin mucormycosis has been reported (13). However, some cases of skin mucormycosis were reported in patients with malignancy (14).

Dermatological manifestations of Wegener’s granulomatosis were reported as purpura, ulcers, nodules, necrotic papules, gingival hyperplasia, pustules, and palpebral xanthoma (15, 16). This wide-spectrum range of skin manifestations would lead to misdiagnosis of infectious cases as well as our presented case. It may be more discouraging especially for the infections such as mucormycosis, which is less seen with skin manifestations. Skin disease can be very invasive locally and go through the skin and subcutaneous tissues into the adjacent muscle and fascia (17–20).

## Conclusion

With consideration of the rare skin manifestation of the mucormycosis in a background history of Wegener’s granulomatosis with cutaneous presentation in current presented case, the authors emphasize the role of definitive differentiation between symptoms of vasculitis diseases such as Wegener’s granulomatosis and those developed as iatrogenic adverse effects of immunosuppressive therapy.

## Ethical considerations

Ethical issues (Including plagiarism, informed consent, misconduct, data fabrication and/or falsification, double publication and/or submission, redundancy, etc.) have been completely observed by the authors.
